# Challenges in control of COVID-19: short doubling time and long delay to effect of interventions

**DOI:** 10.1098/rstb.2020.0264

**Published:** 2021-07-19

**Authors:** Lorenzo Pellis, Francesca Scarabel, Helena B. Stage, Christopher E. Overton, Lauren H. K. Chappell, Elizabeth Fearon, Emma Bennett, Katrina A. Lythgoe, Thomas A. House, Ian Hall, University of Manchester COVID-19 Modelling Group

**Affiliations:** ^1^ Department of Mathematics, The University of Manchester, Manchester, UK; ^2^ Clinical Data Science Unit, Manchester University NHS Foundation Trust, Manchester, UK; ^3^ Joint UNIversities Pandemic and Epidemiological Research, UK; ^4^ The Alan Turing Institute, London, UK; ^5^ LIAM - Laboratory of Industrial and Applied Mathematics, Department of Mathematics and Statistics, York University, Toronto, Ontario, Canada; ^6^ CDLab - Computational Dynamics Laboratory, Department of Mathematics, Computer Science and Physics, University of Udine, Italy; ^7^ Department of Plant Sciences, University of Oxford, UK; ^8^ Big Data Institute, University of Oxford, UK; ^9^ Department of Zoology, University of Oxford, UK; ^10^ Department of Global Health and Development, London School of Hygiene and Tropical Medicine, UK; ^11^ CMMID - Centre for the Mathematical Modelling of Infectious Disease, London School of Hygiene and Tropical Medicine, UK; ^12^ Emergency Response Department, Public Health England, UK; ^13^ IBM Research, Hartree Centre, SciTech Daresbury, Warrington, UK

**Keywords:** early growth rate, unconstrained epidemic, reproduction number, non-pharmaceutical interventions, incubation period, onset-to-hospitalization delay

## Abstract

Early assessments of the growth rate of COVID-19 were subject to significant uncertainty, as expected with limited data and difficulties in case ascertainment, but as cases were recorded in multiple countries, more robust inferences could be made. Using multiple countries, data streams and methods, we estimated that, when unconstrained, European COVID-19 confirmed cases doubled on average every 3 days (range 2.2–4.3 days) and Italian hospital and intensive care unit admissions every 2–3 days; values that are significantly lower than the 5–7 days dominating the early published literature. Furthermore, we showed that the impact of physical distancing interventions was typically not seen until at least 9 days after implementation, during which time confirmed cases could grow eightfold. We argue that such temporal patterns are more critical than precise estimates of the time-insensitive basic reproduction number *R*_0_ for initiating interventions, and that the combination of fast growth and long detection delays explains the struggle in countries' outbreak response better than large values of *R*_0_ alone. One year on from first reporting these results, reproduction numbers continue to dominate the media and public discourse, but robust estimates of unconstrained growth remain essential for planning worst-case scenarios, and detection delays are still key in informing the relaxation and re-implementation of interventions.

This article is part of the theme issue ‘Modelling that shaped the early COVID-19 pandemic response in the UK’.

## 1. Introduction

In December 2019, a cluster of unexplained pneumonia cases in Wuhan, the capital of Hubei province in the People's Republic of China, rapidly progressed into a large-scale outbreak, and a global pandemic by 11 March 2020, as declared by the World Health Organization [1]. The disease caused by this highly contagious infection has since been named COVID-19 and is caused by a single-stranded RNA coronavirus (SARS-CoV-2) similar to the pathogen responsible for severe acute respiratory syndrome (SARS) and Middle East respiratory syndrome (MERS) [2]. As of 29 March 2020, 657 140 confirmed cases and 29 957 deaths have been reported in nearly 200 countries and territories globally [3].

Various control measures have been implemented worldwide, including isolation of confirmed and suspected cases, contact tracing, and physical distancing. In Hubei, a regional lockdown was implemented on 23–24 January 2020, with a peak in reported cases occurring approximately two weeks later [4]. In Italy, a national lockdown was implemented on 9 March 2020 once 7375 confirmed cases and 366 deaths had been recorded, with the epidemic appearing to be slowing down by 29 March [5]. In comparison, India declared a nationwide lockdown on 24 March 2020, with only 434 confirmed cases and 0 deaths [6,7]. Similarly, South Africa began a 21-day lockdown on 27 March, with 927 known cases and 0 deaths [8,9]. Despite the difficulties in evaluating the real extent of these epidemics owing to under-reporting, the implementation of such early and aggressive control measures in India and South Africa may have substantially increased their chances of successful initial containment, notwithstanding the social and personal cost [10].

Besides other indicators like disease severity, fatality rate and hospital occupancy, initial planning of interventions often relies on estimates of the basic reproduction number *R*_0_. This is defined as the average number of new infections generated by a single infected person in a fully susceptible population without control in place and determines the portion of transmission that needs to be prevented to avoid spread [11]. Reported estimates of *R*_0_ for COVID-19 are highly variable, ranging from 1.4 to 6.49 [2,12], with most official sources settling in the range of 2–3 [4,13–16]. However, the latter estimates mostly derive from early studies of the epidemic in Wuhan [17–19] or the Diamond Princess Cruise ship [20], and so are subject to important limitations, including small amounts of data, limited information on epidemiological parameters, uncertain or biased reporting of early cases, and the uniqueness of the specific settings in which they occurred. We argue that continuous effort should be devoted to understanding the discrepancies in published values, and official ranges of *R*_0_ should be continuously updated with estimates coming not only from China [21,22], but also from the many different outbreaks observed worldwide [23–26]. Point estimates might not change, but the task remains imperative both because available data become more numerous and reliable, and because estimates of *R*_0_ in one population do not necessarily translate to another.

However, scientific results should not be summarized solely as *R*_0_ estimates, and policy decisions should not rely exclusively on *R*_0_ as a measure of epidemic spread. First, *R*_0_ lacks temporal information related to the speed of epidemic growth, and hence the optimal timing of interventions [11,27]. Second, estimates of *R*_0_ can vary considerably—and in particular do so for COVID-19—not only as a reflection of genuine differences in geography and settings [11], but also because of how they are calculated: *R*_0_ is typically indirectly derived through mathematical models, with values varying depending on model structure and estimates of, or assumptions on, parameter values (e.g. the generation time distribution or the amount of pre-symptomatic transmission), even when the same data are used for model fits. While numerous estimates of *R*_0_ for COVID-19 exist, real-time growth rates are much less prominent in the literature and, where published, lack robustness or are restricted to single-country analyses on a single dataset [23,28]. We argue that the real-time growth rate and the delay between infection and case detection are often more informative than precise estimates of *R*_0_ for initiating interventions. Unlike *R*_0_, they can inform how quickly cases will reach certain thresholds and hence how long in advance interventions should be introduced to avoid such thresholds being breached. Moreover, because doubling times and delays can be inferred directly from incidence and line-list data, sophisticated models are not required to infer when action is urgent.

We first estimated the growth rate of COVID-19 epidemics, in multiple countries and with different methods, before strong physical distancing interventions were implemented. We then estimated the incubation period and the distribution of times from symptom onset to hospitalization under multiple scenarios and in different settings, and compared it with other results from the literature. Our results show that, in an unconstrained epidemic and in the absence of a robust testing structure, cases can grow eightfold before the effect of any intervention becomes visible. Observation of the initial outbreaks in the UK and Italy confirms these results. Finally, we discuss the limitations of relying solely on *R*_0_ as a measure of epidemic spread.

## 2. Results and discussion

### (a) Unconstrained epidemics in Europe doubled on average every 3 days

**Figure 1.**
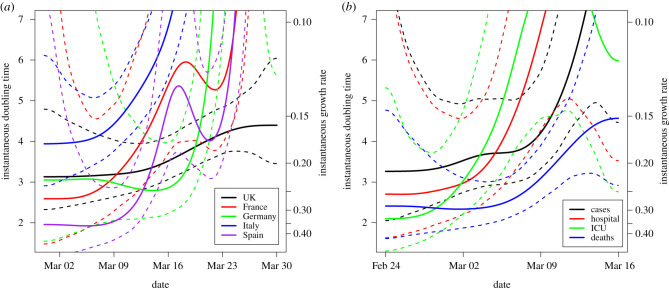
Time-varying doubling time from multiple countries and data streams. Instantaneous doubling time (left axis) and growth rate (right axis), with 95% confidence intervals (CIs, dashed) obtained by fitting a GAM with quasi-Poisson family and canonical link to data, adjusted by day-of-week fixed effect (see the electronic supplementary material), to (*a*) daily confirmed cases of the five largest European epidemics since the beginning of March 2020, and (*b*) different surveillance data streams within Italy since late February 2020. The approximately constant values observed in the early epidemic in each country describe the phase of unconstrained exponential growth, before physical distancing slowed it down. Notice that the apparent 4-day doubling time for confirmed cases in Italy at the beginning of March (*a*) is already higher than the unconstrained doubling time of just over 3 days visible in late February (*b*). Hospital and ICU daily counts were obtained from bed occupancy data to provide a fairer comparison in both point estimate and uncertainty with daily confirmed cases and deaths, under the assumption that hospital and ICU length of stay is long enough that discharges are negligible in the time frame considered.

For the estimation of the growth rate, we focussed on the number of confirmed cases in the five most affected European countries (figure 1*a*), as reported by the World Health Organization [29] on 31 March 2020. To avoid relying only on confirmed cases, which could be affected by numerous biases, we also estimated the growth rate in Italy from additional data streams, specifically hospital and intensive care unit (ICU) bed occupancy and deaths (figure 1*b*; [30]). The analysis accounts for the temporal evolution of the growth rate, with time-varying instantaneous growth rate estimates obtained from a semi-parametric generalized additive model (GAM), and for changes in reporting by day of the week (details in the electronic supplementary material). For all countries and metrics in figure 1, the initial doubling times (at the end of February for Italy and in early March for the other countries, whose epidemics started later) varied between 2 and 3.5 days, with these values increasing over time owing to the introduction of physical distancing.

**Figure 2.**
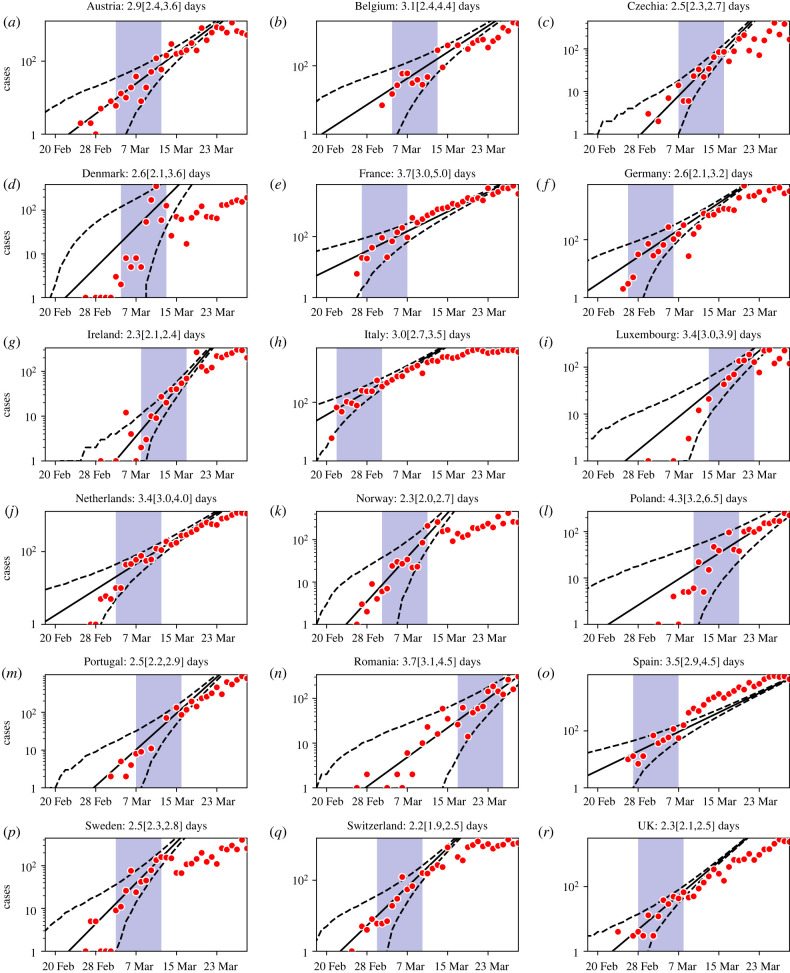
Unconstrained doubling times across Europe. Log daily confirmed cases (red dots) and exponential fit (solid) and 95% CIs (dashed black lines) for all European countries with more than 1000 cumulative confirmed cases by 27 March, obtained using a GLM (see the electronic supplementary material) in the 9-day data period after a cumulative incidence of 20 is reached (shaded area). Slight adjustments to Denmark and Romania reflect their particular circumstances (see the electronic supplementary material). Doubling times with 95% CIs are reported above each panel.

For robustness and to ensure generalizability of results, we performed another analysis on a larger set of European countries (figure 2) using a generalized linear model (GLM). An advantage of the GLM is that the resulting point estimates are easy to interpret as the slope of the line that best fits the data points on a logarithmic scale. However, these estimates are highly sensitive to the choice of the fitting window, especially with small numbers, noisy data, fast growth and rapid implementation of interventions, so we identified criteria for a consistent window choice (figure 2; electronic supplementary material). We estimated mean doubling times before mitigating interventions ranging from 2.2 to 4.3 days, with an average of just under 3 days across all regions (figure 2) and slightly less if three (Denmark, Norway and Poland) to six countries (additionally, Belgium, the Netherlands and Portugal), with less convincing fits, are discarded. These values are significantly shorter than the 6–7-day estimates obtained from China [17,31] that dominated the early published literature. Unsurprisingly, the results obtained with the GAM and the GLM differ in terms of their confidence intervals, but the conclusions are similar and are consistent with a visual inspection of the data plotted on a logarithmic scale (electronic supplementary material, figure S4).

Although our results are robust to the method used, they could still be misleading if there are biases in the data, such as errors in reporting, changes in case definition or testing regime, etc. This issue is particularly critical as lags in reporting of cases can create discrepancies between national and international official sources [29,30,32] for case counts. However, these are unlikely to affect our conclusions owing to the following considerations:

— the fast growth and high numbers probably make small biases negligible;
— any multiplicative correction, such as constant under-reporting, does not affect the observed exponential trend;
— in the absence of increasing community testing, the exponential growth of the underlying epidemic process can easily be underestimated, e.g. if reporting rates decline over time, but is harder to consistently overestimate. Aggressive swabbing of asymptomatic individuals (e.g. early on in the Italian locked-down towns [33]), as well as changes in case definition [34], might explain such a bias in the data we analysed, but are unlikely to affect observations for longer than a few days or consistently across different countries; and
— hospital and ICU bed occupancy, which in the Italian data grow at similar rates as the number of confirmed cases (figure 1*b*), are less affected by reporting issues. The observed faster increase in death rates, instead, may be explained by clustered outbreaks among vulnerable groups (e.g. care home residents) coupled with quicker progression to death among these groups, or possibly local hospital saturation.

We conclude that, although each data stream has limitations, the evidence for fast exponential growth in the absence of intervention is compelling.

### (b) Delay of approximately three doubling times between infection and case detection

Non-pharmaceutical interventions, with unknown adherence, cannot be evaluated until their effects emerge in the data. The delay between infection and case detection is therefore crucial in determining how long the current growth could continue before the slowdown owing to a newly introduced intervention becomes visible. Pre-symptomatic detection is difficult once containment has failed since it relies on extensive contact tracing and testing of asymptomatic individuals. Detecting cases at symptom onset is more feasible, but depends on the case-finding strategy. For example, from 13 March 2020, symptomatic individuals in the UK have been instructed to self-isolate at home and are only tested if they subsequently require hospitalization. Thus, the delay between infection and case detection includes the incubation period, the time between symptom onset and hospitalization, and the time required to receive a positive test result. Similar effects will be visible in other countries where case counts are dominated by hospitalizations.

We report published estimates of the incubation period and the delay between symptom onset and hospitalization (table 1). Because none of these estimates simultaneously account for truncated observations and exponential growth in the number of infected cases, we also include our own estimates (see the electronic supplementary material and [41]) obtained by analysing UK line-list data provided by Public Health England (unfortunately not publicly available) and a publicly available line-list which collates worldwide data [38]. Our estimates are more robust but broadly consistent with the existing literature, and highlight geographical heterogeneity, such as shorter onset-to-hospitalization intervals in Hong Kong and Singapore compared to the UK. With the exception of Singapore, the sum of the mean incubation period and mean onset-to-hospitalization interval is never shorter than 9 days, which corresponds to approximately three doubling times in an unconstrained epidemic like those observed in figures 1 and 2.

**Table 1.** Estimates of incubation period and delays from onset of symptoms to confirmation/hospitalization from the literature and from our analysis (see the electronic supplementary material).

parameter	mean	s.d.	confidence interval for the mean	sample size	source
incubation period	4.8	—	2.2–7.4	16	[35]
incubation period	5.6	3.9	4.4–7.4	52	[36]
incubation period	6.4	2.3	5.6–7.7	88	[37]
incubation period	4.85	2.79	—	162	electronic supplementary material (Data – [38])
onset to confirmation	4.8	3.03	—	38	[39]
onset to hospitalization	5	—	—	—	[40]
onset to hospitalization (dead)	6.6	—	5.2–8.8	34	[36]
onset to hospitalization (alive)	9.7	—	5.4–17	155	[36]
onset to hospitalization (UK)	5.14	4.20	—	90	electronic supplementary material (Data - PHE)
onset to hospitalization (Singapore)	2.62	2.38	—	92	electronic supplementary material (Data - [38])
onset to hospitalization (Hong Kong)	4.41	4.63	—	52	electronic supplementary material (Data - [38])

**Figure 3.**
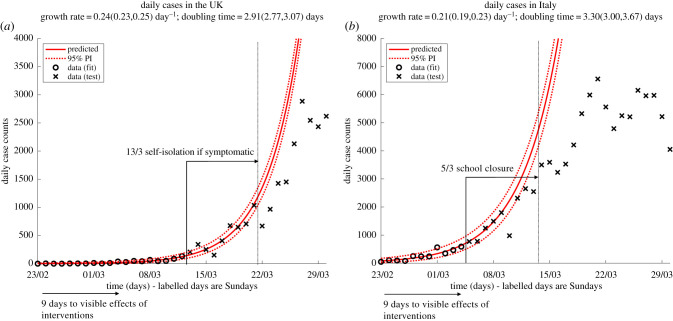
Observed deviation from unconstrained exponential growth approximately 9 days after the first publicly announced nationwide intervention. Daily confirmed cases in (*a*) the UK and (*b*) Italy before intervention (circles) are fitted with a GLM with 95% negative binomial prediction intervals around the central estimate (dashed red lines; see the electronic supplementary material). Crosses are data not used for fitting. Maximum-likelihood estimates of growth rates and doubling times, with asymptotic 95% CIs, are reported above each panel.

Our estimates of doubling time and delay between infection and detection are consistent with observations of the UK and Italian epidemics (figure 3). For both countries, after the first substantial national-scale intervention was implemented, the number of daily confirmed cases sustained the pre-intervention exponential growth for about 9 days, before deviating from the predicted trajectory. After the first control measure in the UK (recommended self-isolation if symptomatic from 13 March, figure 3*a*), cases continued to increase exponentially with an estimated growth rate of approximately 0.24 d^−1^ (corresponding to a doubling time of just under 3 days) for 9 days. During this period the number of daily confirmed cases rose approximately eightfold. A similar pattern was observed in Italy (nationwide school closure imposed on 5 March, although local lockdowns were imposed and universities in Northern Italy were closed between 22 and 23 February, figure 3*b*).

Owing to the delay between implementing interventions and observing their effects in the data, a pattern of introductions of increasingly strong measures has repeated across Europe, with long delays to control. Even with immediate hard interventions halting all community transmission, within-household transmission will continue to occur, creating an additional delay between the implementation of the intervention and observing its effect. This is consistent with the approximate two-week delay from lockdown to peak in new cases observed in Hubei [4]. Further delays in hospital/ICU bed occupancy and deaths mean the latter figures keep on growing well after transmission control is achieved.

### (c) *R*_0_ estimates quoted in isolation have limited value

Because *R*_0_ remains the mainstay of most epidemiological analyses, we explored values of *R*_0_ consistent with a range of growth rates and modelling assumptions. For a growth rate of 0.25 d^−1^ and our estimates of the incubation period (table 1), we obtained values ranging from 2 to 5 (electronic supplementary material, table S1*a*), owing to the extreme sensitivity to assumptions, in particular the extent of pre-symptomatic transmission, for which estimates in the literature vary widely [42–45]. Conversely, the same *R*_0_ can be derived from different growth rates, if different generation times are assumed. For this reason, *R*_0_ values can be misleading if quoted in isolation: for example, a value of *R*_0_ of 2.2 was estimated in [31] from a doubling time of 7.4 days and relatively long SARS-based generation times (assumed in the absence of reliable information), but the same numerical value could be obtained with a shorter and more front-loaded infectivity profile (e.g. from electronic supplementary material, table S1*a*, gamma-shaped infectivity with mean 2 days and 2 days of pre-symptomatic transmission) and an epidemic that is growing more than twice as fast (e.g. from electronic supplementary material, table S1*a*, a growth rate of 0.2 d^−1^, corresponding to a doubling time of 3.47 days).

Although *R*_0_ determines the reduction in person-to-person transmission needed to achieve control, this brings limited insight into the early response to COVID-19. The challenge in measuring the amount of pre-symptomatic transmission translates into uncertain quantification of most likely *R*_0_ values even when derived from the same growth rate (electronic supplementary material, table S1*a*). Moreover, with substantial pre-symptomatic transmission, the exact value of *R*_0_ only poorly correlates with the feasibility of infection control [46]. For example, if *R*_0_ = 4 and all transmission occurs after symptoms onset, self-isolation when symptomatic can achieve the required 75% fall in transmission. Conversely, if *R*_0_ = 2 but most transmission is pre-symptomatic, unless a solid infrastructure is in place to rapidly detect and isolate pre-symptomatic individuals, the required 50% drop in transmission can only be achieved through interventions, like mass quarantine of apparently healthy individuals, that are highly socially disruptive and probably enforced rather than spontaneous. Finally, *R*_0_ informs how much transmission should be averted, but not how quickly this should be achieved.

Ultimately, neither *R*_0_ nor the growth rate can inform the choice of intervention, because of uncertainty in the nature and proximity of contacts necessary for transmission, the challenges in quantifying the risk of transmission through indirect routes (e.g. fomites) and, not least, unpredictable levels of adherence. Such uncertainties on the impact of behavioural changes, further coupled with extremely variable *R*_0_ estimates, probably result in the need for a major reassessment of intervention strategies after their implementation. With long delays to observing the effect of interventions and a concurrent fast growth, proper, data-driven, reassessment is feasible only after a long enough period of time, during which detected cases can grow substantially (figure 3). If, at that point, more stringent measures are necessary, they will require further time to show an effect, while cases continue to grow. Immediate action, when cases are low, can grant enough margin for informed reassessment without overwhelming the healthcare system. In planning interventions, growth rate and delays to detection are more suitable than *R*_0_ to provide guidance on such temporal details.

## 3. Conclusion

The highlighted risks of underestimating the combination of short doubling times and long delays between infection and case detection are consistent with the now-common pattern of countries misjudging the initial small number of observed cases, only to realize the storm has already arrived. With cases largely being confirmed only when hospitalized, after the introduction of interventions, epidemic growth will be sustained for at least a full infection-to-hospitalization delay before their effect is observed. With unconstrained growth, our estimates mean that cases can grow more than eightfold during this period and, if the healthcare system risks being overwhelmed, even the extreme effort of doubling hospital capacity only buys 3 days of reprieve. Although *R*_0_ is valuable for quantifying the fraction of transmission that should be prevented to control the epidemic, estimates of *R*_0_ reported or quoted in isolation can be misleading, in particular when similar values are obtained from very different growth rates and modelling assumptions. This issue is particularly relevant in the case of COVID-19, where scientific and media communications overly focussed on *R*_0_ may have contributed in distracting the attention from epidemic growth with doubling times that were significantly shorter than those dominating the early published literature. We advocate stronger action from national and international healthcare communities, with a particular focus on supporting low- and middle-income countries where numbers of cases, at the time of writing (end of March 2020), appeared to be relatively low. In settings where healthcare capacity is low and intergenerational mixing common, swift action can save numerous lives.

## Addendum

Our original analysis was uploaded as open-access preprints on 31 March [47] and 15 April 2020 [48]. The *medRxiv* version [48] was updated on 11 June 2020 to reflect comments from reviewers, including revised figures, concepts clarification and a discussion update to make the paper more relevant to the situation in June. In line with the spirit of this special issue collating analyses that have shaped COVID-19 policy in the UK, here we have tried to remain as close as possible to the sentiments expressed in the original version, including the call for action we advocated at the time. However, we have retained the revised analysis from [48] (plus a further correction to figure 1*b*) and updated the original preprint citations to peer-reviewed published versions where appropriate. The conclusions have not changed throughout.

Since our original preprint, evidence has emerged confirming the link between early interventions and lower mortality [49–51], and our statement that mass quarantining would be followed by a wave of within-household transmission has later been verified [52].

**Figure 4.**
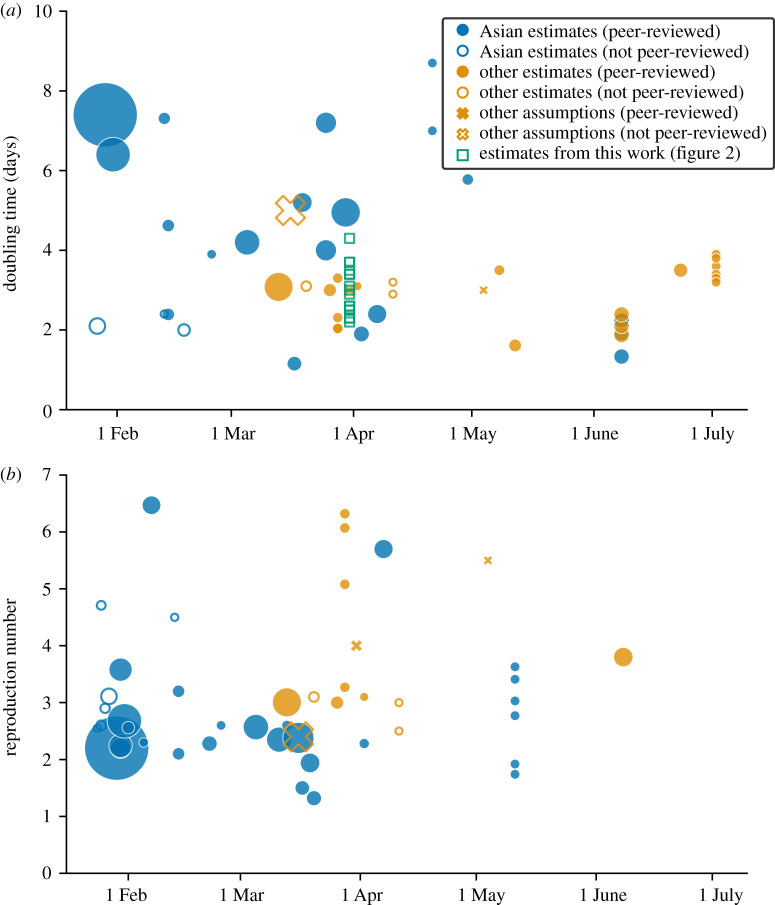
Estimates of (*a*) unconstrained (or early) doubling time, and (*b*) basic (or early) reproduction number published in the first half of 2020, by date of publication. The size of the marker indicates the number of Google Scholar citations recorded between 31 March and 1 April 2021. Publications with fewer than 100 citations are not shown, with the exception of the estimates presented in this paper (green squares). Filled markers denote peer-reviewed publications and empty markers preprints or other reports. Circle markers denote estimates obtained from data (whether made available or not) and crosses are values presented as assumptions or statements with no clear source. Blue colour denotes publications using data from, or presenting results for, countries in Asia, while yellow is used for all other countries. Publications have been extracted from Google Scholar between 31 March and 1 April 2021, with searches [(‘doubling time’ OR ‘growth rate’) AND (covid OR SARS-CoV-2)] and [‘basic reproduction number’ AND (covid OR SARS-CoV-2)]. The resulting filtered list is available at https://github.com/thomasallanhouse/covid19-growth. Longer doubling time estimates and low reproduction numbers dominated the cited literature at the time of first submission (a citation count collected on 18 April 2020 for the most cited publications in this figure resulted in fewer citations, but in broadly similar proportions, thus providing the same qualitative picture—not shown), and still dominate it now. Although longer doubling times observed in Asia could in principle be attributable to a different variant [53], short doubling times were also obtained from China, both early on (e.g. in January 2020 by Read *et al*. [54], also published in this Special Issue) and in later studies re-analysing early data [41,55].

Despite a broad range of initial doubling time estimates reported between January and March of 2020, early estimates [17,31] or assumptions [40] at the higher end of the spectrum (5–7 days) dominated the literature at the time of submission and still dominate it today (figure 4). The limited duration of the period of unconstrained growth and the noisiness of data collected early on in each country call for a robust analysis making use of multiple data streams (in particular, hospital and ICU data, which are less affected by reporting biases than confirmed cases) and comparing multiple countries. With very few exceptions [55–57], little work has since been published with levels of robustness similar to those in this study. However, highly visible robust analyses are essential, both owing to their direct importance for informing government response, but also because crucial work on e.g. severity estimates, scenario planning, and control policies using the early estimates is still being cited.

In this study, we also highlighted potential problems arising from using *R*_0_ estimates in isolation, or comparing them directly between different studies. The published literature has been awash with estimates of *R*_0_, obtained from a plethora of data sources and often subsequently cited without discussion of the underpinning technical details and assumptions. In addition to the data and method of choice, estimates of *R*_0_ are sensitive to assumptions on the serial interval, generation time and pre-symptomatic transmission [42,43,58,59]. The broad range of estimates, from 0.48 to 14.8 [60], underscores the magnitude of the problem. The fact that peer-reviewed publications discussing these details have only become available towards the end of 2020 [61] highlights the limited attention this topic has received.

Even though our analysis focussed on the early stage of the pandemic, the conclusions remain important. First, our estimates of the unconstrained growth rate are essential to plan worst-case scenarios and calibrate models guiding both the relaxation and the re-implementation of interventions. Second, recognizing detection delays can help to avoid overconfidence in epidemic control when interventions are relaxed: new infections can build up unnoticed for several days while detected cases appear to be consistently decreasing. Similarly, when interventions are tightened, there will be a delay between the new control policies and seeing their effect in the data. Ideally, delays should be minimized by implementing significant levels of community testing of asymptomatic as well as symptomatic individuals, and intervals between changes in interventions should be long enough to allow for assessment of their impact.

A final key message of our work is the unnecessary predominance of reproduction numbers in the scientific and public discourse. Although growth rate estimates are now regularly published [62] and visualized [63], reproduction numbers still appear to receive the highest emphasis from public health officials, policy makers and the media. For the most part, estimates of the current reproduction number are used to assess whether new interventions are having the desired effect (i.e. reproduction numbers are decreasing over time) or whether local or national epidemics are brought under control (i.e. reproduction numbers are falling below one), even though the same signal could be read by just observing whether the data are growing more slowly over time, or are reversing their trend from growth to decay.

In conclusion, COVID-19 short doubling times highlight the importance of careful evaluation of time indicators together with the time-insensitive reproduction numbers. These aspects cannot be underestimated especially in the context of new, unknown, emerging infectious diseases and high uncertainty in parameter values and reproduction number estimates. Besides the speed of growth, a careful evaluation of infection-to-detection delays is crucial for planning the implementation and relaxation of non-pharmaceutical interventions, to anticipate when they will take effect and allow for margins to measure their impact and, if needed, re-asses planned strategies.

## In context

### How this analysis shaped policy

The first key result in this paper, namely that numbers of confirmed COVID-19 cases in the UK were doubling approximately every 3 days, with similar rates in other European countries (specifically, figure S4 in the electronic supplementary material and a preliminary version of figure 3*a*), was presented to the Scientific Pandemic Influenza group on Modelling (SPI-M) on 20 March 2020.

The context in which these estimates were made is illustrated in figure 4. At the time, estimates of the basic reproduction number and the unconstrained growth rate of SARS-CoV-2, obtained predominantly using data from China, varied widely. However, some of these appeared only in non-peer-reviewed preprints (including those from Read *et al.* [54], published in this issue), with the discourse dominated by estimates coming from the most established epidemic modelling groups and the highest-impact journals. In line with these, the consensus of the Scientific Advisory Group for Emergencies (SAGE) at the time was on a doubling time of around 5–6 days^1,2^ and a UK epidemic lagging 2–4 weeks behind the Italian one^3^.

Our estimates were not the only ones presented at the SPI-M meeting on 20 March: a preliminary analysis from Public Health England's Joint Modelling Team, based on the then scarce and rapidly changing confirmed cases and deaths in England, also estimated a 3-day doubling time. What made our analysis particularly robust was the comparison between confirmed cases from multiple countries with similar social structures to the UK and the use of hospital and ICU data from Italy, as these data streams suffer from fewer biases compared to confirmed cases and deaths (see §2). With two independent analyses pointing in the same direction at the meeting, SAGE's consensus estimates were rapidly revised. A second analysis presented at the same meeting by the authors additionally evidenced that the UK epidemic was lagged by two weeks compared to the Italian one, firmly at the low end of the SAGE's consensus range at the time.

Although the pressure for further interventions was mounting and a lockdown was becoming unavoidable, awareness of the speed of spread provided key evidence in the decision-making process that led to the closure of pubs and restaurants the same day.

At the SPI-M meeting on 23 March 2020, we presented a report detailing the second key result in this paper, namely the unavoidable delay of at least 9 days between interventions and observing their effect in the data. Coupled with the 3-day doubling time, this meant that, even with immediate intervention, hospital and ICU bed projections would likely pass the target limits set out by the NHS at the time. These analyses made it clear that decisions had to be taken immediately, before assessing the effect of the previous closure of hospitality venues, thus providing key evidence supporting the first national lockdown coming into force the next day.

### Struggles with peer-review publication process

Given the international importance of our results, we temporarily interrupted the modelling work in support of the UK response and instead aimed for rapid, peer-reviewed, publication [47]. The choice of the journal was not straightforward, and particularly acute was the awareness that the race for first publication and for highest impact journals was at least partially responsible for the dominance of longer doubling time estimates in the cited literature. Nevertheless, the assurance of rapid review, immediate sharing of the submitted manuscript with the WHO, and consequently the potential for our results to support a decisive pandemic response in other countries, were the deciding factors for submission to a high-impact journal. Unfortunately, the review process took a month, and the paper was rejected as the results, albeit valued as scientifically robust, were perceived to be insufficiently novel and not changing the understanding of SARS-CoV-2 spread at the time.

Although we agreed that by April 2020 an expert in the field might not have been surprised by the main results of the paper, we appealed on the basis that (i) the key messages, together with the discussion of the relevant merits of *R*_0_ and the growth rate as indicators of viral spread, were of continued importance for policy makers and general audiences, especially in a context of rapidly changing interventions, and (ii) the publication offered robust multi-country, multi-data-stream estimates, and hence filled a gap in the referenceable literature. After an invitation to resubmit, we re-drafted the paper in light of the new prevailing questions faced by governments worldwide, highlighting the relevance of our estimates for relaxing non-pharmaceutical interventions while preventing cases from accumulating unnoticed ([48]; see Addendum). The manuscript was again rejected based on it being outdated and the inconsistency of discussing reopening while analysing data about the unconstrained growth phase.

Our experience epitomizes some of the key issues associated with the need for rapid but rigorous scientific publishing in an emergency. Many of these issues have been experienced by the authors of other papers in this Special Issue, raising important questions:

— How rigorous should a scientific analysis be under time constraints? Rapid analyses can be done – the fast growth in cases in the UK and abroad in March 2020 was clear from simple data visualizations—yet governments may be unwilling to act based on non-peer-reviewed results. A publishable and rigorous scientific analysis, complete with uncertainty quantification and discussion of the potential biases, requires time, but if the results are similar to those already in the public domain they may be judged to be insufficiently novel for publication.
— When should a scientific result be considered ‘well known’ and hence not worth publishing? In a pandemic, understanding of the situation can change rapidly, and the situation itself will likely differ by regions. Results that one person (such as an expert reviewer sitting on the same committees) may be aware of, could be of extreme interest and importance to others.
— To whom should scientific publications be targeted? Straightforward arguments expressing key concepts could be extremely useful for policy makers, but not novel to experts. Conversely, complex analyses might be scientifically novel but not offer sufficient clarity upon which policy makers can act.
— How can timely, visible, publication be ensured? The perceived value of a potential publication is by definition subjective, and reviewers can effectively block publication on this basis. Under normal circumstances, a rejection is disappointing, and typically leads to re-submission to a different journal. However, in the context of a rapidly changing pandemic situation, a rejection delays the utility of the research and could render it effectively unpublishable. For example, modelling investigations used to explore scenarios and inform policy decisions in the absence of data, though of general interest, are bound to become outdated once more data are available or the decisions they contributed to have been taken. Furthermore, the same researchers may have limited capacity to revise and resubmit previous research as the attention shifts to new policy asks.

These issues are particularly acute when transparency in the scientific evidence leading to policy choices is demanded by the public, but scientific publications and citation counts, rather than policy impact, ultimately drive the award of research funding—including short-term funding needed to respond to the crisis. Processes enabling rapid peer-review have been proposed^4^ and trialled^5^ [64], but are currently in their infancy. Alternative publication models, based on immediate online access and transparent peer review (e.g. F1000Research, Wellcome Open Research, eLife), that could partially address these problems also exist, but are not yet widespread.

### The critical role of early estimates and their revision

By placing our paper in context, we also hope to raise awareness of the risks associated with the need for rapid dissemination of results based on limited data. In particular, early estimates published in high-impact journals can have high visibility that is amplified as later studies refer to them. If erroneous—which is more likely in a system where the reward is skewed in favour of being the first published estimates, sometimes at the cost of scientific robustness—these estimates can have substantial negative consequences for policy decisions. Clear communication of uncertainty, unavoidable with limited early data, is crucial and should be aided when possible by baseline agreed-upon standards and protocols for ‘week 0’ data analysis. Similarly, increased data availability and transparency should enable critical and constructive cross-checking of results by different research groups.

A system that rewards confirmatory or updated analyses and the gathering of robust scientific evidence, and that supports the scientists doing this work, should be a fundamental principle in future pandemics and public emergencies.

## Data accessibility

All data and code used in this analysis are provided in the Github repository https://github.com/thomasallanhouse/covid19-growth, with the exception of the UK line-list data used to estimate the onset-to-hospitalisation delay, which are provided by Public Health England under a data-sharing agreement and not publicly available.

## Endnotes

^1^See https://assets.publishing.service.gov.uk/government/uploads/system/uploads/attachment_data/file/873713/01-spi-m-o-consensus-statement-on-2019-novel-coronavirus-_covid-19_.pdf.

^2^See https://assets.publishing.service.gov.uk/government/uploads/system/uploads/attachment_data/file/888784/S0384_Sixteenth_SAGE_meeting_on_Wuhan_Coronavirus__Covid-19__.pdf.

^3^See https://assets.publishing.service.gov.uk/government/uploads/system/uploads/attachment_data/file/888785/S0385_Seventeenth_SAGE_meeting_on_Covid-19_.pdf.

^4^See https://royalsociety.org/topics-policy/Health%20and%20wellbeing/ramp/.

^5^See https://gateway.newton.ac.uk/news/2021–02-10/10378.
